# Examining the Visual Search Behaviour of Experts When Screening for the Presence of Diabetic Retinopathy in Fundus Images

**DOI:** 10.3390/jcm14093046

**Published:** 2025-04-28

**Authors:** Timothy I. Murphy, James A. Armitage, Larry A. Abel, Peter van Wijngaarden, Amanda G. Douglass

**Affiliations:** 1 School of Medicine (Optometry), Faculty of Health, Deakin University, Geelong, VIC 3216, Australia; tim.murphy@canberra.edu.au (T.I.M.); j.armitage@deakin.edu.au (J.A.A.); larry.abel@deakin.edu.au (L.A.A.); 2 Faculty of Health, School of Health Sciences, University of Canberra, Canberra, ACT 2601, Australia; 3 Centre for Eye Research Australia, Royal Victorian Eye and Ear Hospital, Melbourne, VIC 3002, Australia; peterv@unimelb.edu.au; 4 Ophthalmology, Department of Surgery, The University of Melbourne, East Melbourne, VIC 3002, Australia; 5 Department of Optometry and Vision Sciences, The University of Melbourne, Parkville, VIC 3010, Australia

**Keywords:** diabetic retinopathy, eye tracking, eye movements, visual search, screening, search strategy, optometrist, ophthalmologist

## Abstract

**Objectives:** This study investigated the visual search behaviour of optometrists and fellowship-trained ophthalmologists when screening for diabetic retinopathy in retinal photographs. **Methods:** Participants assessed and graded retinal photographs on a computer screen while a Gazepoint GP3 HD eye tracker recorded their eye movements. Areas of interest were derived from the raw data using Hidden Markov modelling. Fixation strings were extracted by matching raw fixation data to areas of interest and resolving ambiguities with graph search algorithms. Fixation strings were clustered using Affinity Propagation to determine search behaviours characteristic of the correct and incorrect response groups. **Results:** A total of 23 participants (15 optometrists and 8 ophthalmologists) completed the grading task, with each assessing 20 images. Visual search behaviour differed between correct and incorrect responses, with data suggesting correct responses followed a visual search strategy incorporating the optic disc, macula, superior arcade, and inferior arcade as areas of interest. Data from incorrect responses suggest search behaviour driven by saliency or a search pattern unrelated to anatomical landmarks. Referable diabetic retinopathy was correctly identified in 86% of cases. Grader accuracy was 64.8% with good inter-grader agreement (α = 0.818). **Conclusions:** Our study suggests that a structured visual search strategy is correlated with higher accuracy when assessing retinal photographs for diabetic retinopathy. Referable diabetic retinopathy is detected at high rates; however, there is disagreement between clinicians when determining a precise severity grade.

## 1. Introduction

Diabetes mellitus is a major global health concern, with an estimated 10.5% of the adult population affected by the disease in 2021 [1]. By 2045, an estimated 785 million people will have diabetes [1]. A common complication of diabetes, and one of the most prevalent causes of preventable blindness, is diabetic retinopathy [1,2,3].

While diabetic retinopathy can lead to irreversible vision loss, early forms of retinopathy do not require treatment by an ophthalmologist, and efficient public health systems recommend review and monitoring by optometrists. Several grading systems have been created based on the presence, location, and number of diabetic retinopathy features observed in the retina, with each grade corresponding to a risk of serious complications [4,5]. This guides clinicians in determining treatment and recall schedules [4,5]. Clinical guidelines also exist regarding screening frequency and criteria for referral [4,6,7,8,9]. Despite this, disagreement exists between clinicians when determining the severity of diabetic retinopathy [10,11,12,13,14,15]. While Sellahewa et al. [12] found disagreement was highest for cases of little or no retinopathy, Sallam et al. [15] noted disagreement across all severity grades leading to overall moderate inter-grader agreement. Incorrect grading can result in delayed treatment and potentially lead to vision loss.

It may be possible to reduce inter-grader variability by using a systematic approach to screening. This has proven successful in glaucoma diagnosis and management, with clinicians using the FORGE guidelines [16] showing an improvement in diagnostic performance [17]. A systematic visual search has also shown to be associated with accuracy in radiology [18], dentistry [19], dermatology [20], and pathology [21]. To date, no visual search guidelines have been developed for diabetic retinopathy screening in a clinical setting. Lesions associated with diabetic retinopathy may be found throughout the retina [22,23,24], making it difficult to determine the areas of highest value in terms of screening for the presence or absence of disease. Hence, a systematic approach based on anatomical regions or landmarks may be beneficial.

One method of characterising the visual search strategies of experts is to examine their eye movements as they examine retinal photographs for the presence of diabetic retinopathy. This technique can enable the extraction of fixation patterns to elicit gaze behaviour that is associated with high diagnostic performance. Gaze behaviour is controlled by top–down and bottom–up mechanisms [25,26]. In top–down or “goal-driven” processing, eye movements are shaped by the participant’s ability to exert cognitive control over the search strategy and influenced by attributes such as education and experience, whereas bottom–up or “stimulus-driven” processing is directed by visual saliency and other characteristics of the stimuli. These two distinct processing mechanisms are not mutually exclusive and may both contribute to the overall gaze behaviour [26]; however, bottom–up processing appears to be less used by those with greater experience when searching for a target in an unknown scene [27], and features that are visually salient do not always offer the highest clinical prognostic value.

Eye tracking studies have been performed on glaucoma specialists and ophthalmology trainees when assessing the optic nerve in fundus photographs [28]. The accuracy was higher amongst specialists overall, with gaze patterns suggesting top–down processing for specialists and bottom–up processing for trainees. Similar work has been published by Rangrej et al. [29] in diabetic retinopathy, looking at the gaze behaviours of experts while assessing fundus photos as being normal (no diabetic retinopathy) or abnormal (having features of diabetic retinopathy). While the study could not determine whether top–down or bottom–up mechanisms were at play, a higher accuracy was correlated with an increased dwell time and shorter overall “track length”, defined as the pixel length of the scan path on the image. A higher accuracy was also positively correlated with experience.

This body of work suggests that visual search behaviours are correlated with diagnostic accuracy in tasks involving the assessment of medical images for signs of pathology. While it may be possible to instruct clinicians on the search patterns that they should use, work by Kok et al. [30] shows that this must be paired with knowledge of the underlying condition—the “why”—in order to improve clinical performance. Hence, an intervention looking to change visual search behaviours should also involve a targeted educational component.

Our study expands on the work by Rangrej et al. [29] to examine eye movement behaviour when grading fundus photos across the spectrum of retinopathy severity using the International Clinical Diabetic Retinopathy Severity Scale [4]. Our study also investigates the zoom behaviour of participants and extracts fixation strings associated with correct grading. This study focuses on diabetic retinopathy screening from macular-centred posterior pole fundus photographs due to the widespread use of retinal cameras and their use by both eye health clinicians and allied health professionals in diabetic retinopathy screening programmes [31]. Our results show a correlation between a structured visual search and accuracy in diabetic retinopathy grading. These data also show a tendency to move between four anatomical landmarks when assessing the posterior pole: the optic disc, macula, and superior and inferior temporal retinal arcades. This information provides visual search information that can be paired with relevant pathophysiology education to create a visual search strategy for diabetic retinopathy, in a similar manner to the FORGE guidelines [16].

## 2. Materials and Methods

Participant eye movements were recorded using a Gazepoint GP3 HD at 150 Hz (Gazepoint, Vancouver, BC, Canada) and Philips 243B9/75 23.8-inch flat screen monitor (Philips, Amsterdam, The Netherlands) at a native resolution of 1920 × 1080. Custom software (version 1.0.0) was developed to interface with the Gazepoint to allow additional functionality (described below).

Images were chosen from the publicly available DDR dataset [32], a collection of 13,673 retinal photographs of the posterior pole from numerous devices and fields of view. Two study datasets were created, each containing four images representing each of the five disease severity grades (twenty images in total) per the International Clinical Diabetic Retinopathy Disease Severity Scale [4]. An additional set of 21 assorted images was created as a practice set to allow users to familiarise themselves with the software before starting the study task. Additional details are provided in the Supplementary Materials.

### 2.1. Software

**Figure 1:**
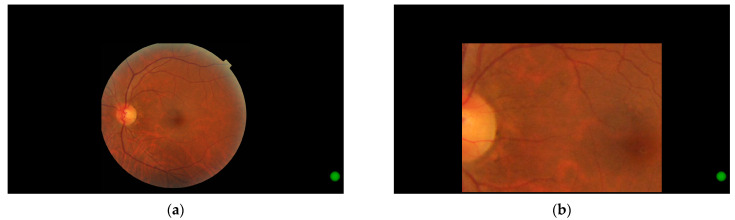
Images as shown to the participant, with the green circle clicked by the user to progress to the grading screen. (**a**) Image at native zoom. (**b**) Zoomed image, staying within the defined border. The software continuously tracked eye movements throughout the dynamic zooming process.

Images were scaled to 1140 × 848 pixels, with original images shrunk to fit within this area and padded with black pixels as required. Each image was displayed at this resolution with a black border of 390 pixels to the left and right, 222 pixels above, and 10 pixels below, as this region has been shown to have the highest accuracy and precision for this eye tracker and monitor combination [33]. Custom software was created to allow participants to pan and zoom within this 1140 × 848 pixel region, as shown in Figure 1, to allow the closer inspection of the image while retaining the relative gaze position on the image and to mimic the typical user experience when performing photographic image grading. All software for image processing and statistical analysis are available at https://github.com/tim-murphy/expert_dr_study (accessed on 10 June 2024).

### 2.2. Participants

Ethical approval was granted by Deakin University Health Ethics Advisory Group (reference: HEAG-H 148_2022). Optometrists and ophthalmologists with vitreoretinal or medical retinal sub-specialist training were recruited from Victoria and New South Wales in Australia. Subjects were contacted directly via email or in person and invited to participate in the study.

The task was undertaken in a dark, quiet room at the participant’s place of work or at the Deakin University campus. After completing a short questionnaire, participants were given a copy of the International Clinical Diabetic Retinopathy Disease Severity Scale [4] and placed approximately 60 cm from the monitor. Participants were told that they would be presented with retinal photographs taken from people diagnosed with diabetes mellitus, but no other patient information was given. When ready, they could progress to the next screen to provide diabetic retinopathy and diabetic macular oedema gradings for the image. Images could be marked as “ungradable” for diabetic retinopathy and/or diabetic macular oedema if the participant deemed the image quality to be too poor for assessment. Participants could not return to the image once they had progressed to the grading screen.

After becoming familiar with the task and software using the practice images, participants were randomly assigned to one of the study’s two datasets and asked to undertake the grading task, with images presented in a random order. Gaze position, cursor position, and zoom information were recorded for each image. Participants were informed that the study was examining correlations between eye movements and grading performance and therefore their eye movement and grading data would be recorded throughout the task. No time limits were enforced, but the time taken to come to a decision was recorded.

### 2.3. Grading Analysis

Each of the images used in the study was paired with a grading decided by majority vote from four registered ophthalmologists, with an experienced specialist acting as arbiter [32]. Grades chosen by participants were compared to these provided grades and converted to a score, with, for example, −1 denoting a grade one level less severe than the provided grade, +2, denoting two grades more severe than the provided grade, and 0 denoting a grade that matched the provided grade. Reponses were then assessed using three methods to provide a more clinical context:

1. A score of 0 was considered correct, with all others considered incorrect;
2. Scores of 0 or 1 were considered correct, with all other responses regarded as incorrect;
3. Images and responses were converted to referable and not referable bins, with referable defined as more than mild non-proliferative diabetic retinopathy, in keeping with other studies [34]. Scores were then calculated using this modified criterion.

The three criteria provide a means of assessing grading accuracy with respect to clinical significance; grading one level higher may trigger unnecessary referrals or financial costs but will not usually result in negative healthcare outcomes. Similarly, identifying when retinopathy requires referral is relevant to patient outcomes, as it reflects how often patients are referred for, or receive, treatment.

Accuracy between groups was compared using Mann–Whitney U tests, as the data were not normally distributed. Krippendorff’s Alpha was used to test inter-grader agreement [35]. Data distribution was assessed using the Shapiro–Wilk test.

### 2.4. Zoom Behaviour Analysis

Zoom and pan functionality was included in the image viewing software to mimic clinical practice. To allow gaze analysis as if using a static image, the zoom level and coordinates of the visible region were appended to all raw gaze data. These data were then modified as follows:

1. Measured gaze position was converted to image pixel coordinates within the visible region.
2. Corresponding coordinates on the original image were calculated from this position.
3. Gaze position in the raw data was overwritten with these new coordinates.

For example, if a gaze position was recorded in the middle of a 50 × 50 region whose top-left corner was at (200, 100), then this would be converted to position (225, 125) on the original image. This allowed gaze data to be analysed without the need for dynamic areas of interest.

These data were then stored in a text file. During post-processing, all data within 200 ms of a zoom change were invalidated, as saccadic eye movements to re-foveate following the change have a latency of at least this duration [36]. Valid gaze positions were then extracted and processed as described below. Zoom metrics were also extracted, with the portion of time spent at each zoom level compared using Mann–Whitney U testing.

### 2.5. Eye Movement Analysis

Analysis was performed on eye tracking metrics for correct and incorrect groups. The total time, fixation counts for each area of interest (AOI), number of visits to each AOI, dwell time per AOI, and image zoom parameters were compared using Mann–Whitney U testing. To find common search patterns, data in each group were clustered using Affinity Propagation [37], using the Levenshtein distance as the distance between results [38]. The largest clusters from each group were then extracted as common patterns.

Areas of interest were derived using Hidden Markov Models (HMMs) [25,39]. All recorded gaze positions were clustered using HMMs and overlayed onto the original images, where they could be visually associated with anatomical features. Features common to most HMMs were deemed AOIs. Each image was then annotated with the pixel locations of each AOI. The Gazepoint GP3 HD accuracy in this screen region is 1.59 degrees [33], which equates to 70 pixels for this monitor at a distance of 60 cm. To account for this, each annotated pixel was expanded to a circle with a 70-pixel radius, meaning that any gaze position recorded within this expanded region could be considered to align with the AOI.

**Figure 2:**
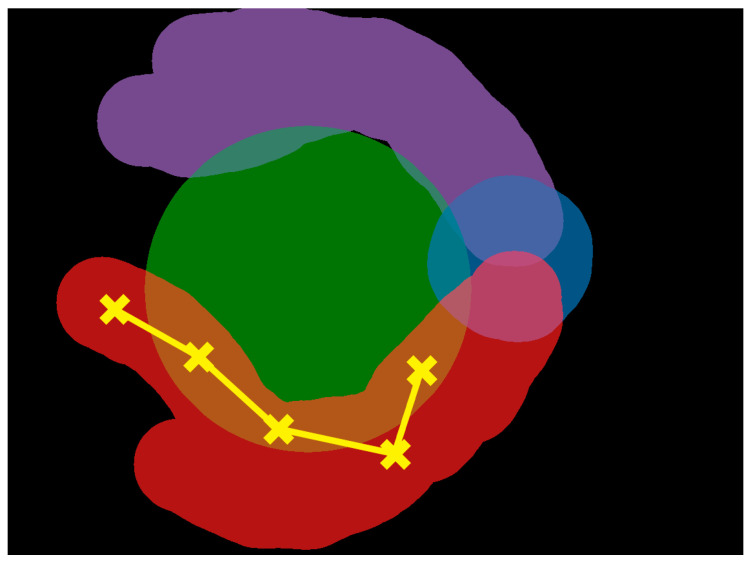
An example scan path showing five fixations: two clearly in the red AOI and three falling in either the red or green AOI. Using the directed acyclic graph method, the ambiguous fixations are associated with the red AOI, as well. AOI, area of interest. Different colours indicate distinct AOIs, with green representing the macula, blue representing the optic disc, purple representing the superior temporal arcade, and red representing the inferior temporal arcade.

To account for overlapping AOI annotations, fixation strings were determined by creating a directed acyclic graph of fixations. Where a single fixation was associated with multiple AOIs, all relevant AOIs were added to the graph at the same level. Each edge was assigned a cost of 1 when joining vertices of different AOIs or 0 if joining vertices of the same AOI. The shortest path was then determined using depth-first search and combining adjacent vertices of the same AOI. A visualisation of this can be seen in Figure 2, where three of the recorded fixations fall within the boundaries of two AOIs (the overlap of red and green AOIs). Given that the two other fixations are clearly in the red AOI, this technique assigns the ambiguous fixations to the red, as well.

To allow a comparison with work by Rangrej et al. [29], the total track length was also calculated for each response. This was calculated as the sum of the distances between successive fixations, in pixels.

Although grades for both diabetic retinopathy and diabetic macular oedema were collected, only the diabetic retinopathy grades were evaluated. Grading macular oedema from a monocular fundus photograph can be unreliable [40], and no oedema grades were included in the image dataset, so it was not possible to assess grading accuracy. This grading task was included for completeness, however, to mimic a diabetes grading task in a clinical setting.

## 3. Results

Data were collected from 23 participants: 15 optometrists and 8 ophthalmologists with sub-specialty fellowship training in medical retina or vitreoretinal surgery. Ophthalmologists were older than optometrists (U = 16.0, *p* < 0.01), although the value for years of clinical experience was not significantly different between groups (U = 34.5, *p* = 0.10). Participant information is shown in Table 1.

### 3.1. Grading Accuracy

Considering all data for all participants, the distribution of scores excluding “ungradable” did not follow a normal distribution (W = 0.797, *p* < 0.01) but was centred around zero, with 64.8% of responses matching the grades in the dataset. However, when considering grade scores of 0 and 1 to be correct, this increased to 80.9%. Images were regarded as ungradable 0.7% of the time (*n* = 3). When considering grading as referable and not referable, the accuracy increased to 86.3%. Similar trends were seen when assessing distribution by image grade, with most responses matching the grade in the dataset. The grade with the lowest agreement was moderate NPDR with 37.5% having a grade score of 0 and 26.4% having a grade score of −1. In all cases, ungradable was considered an incorrect response as the authors considered all images to be of sufficient quality to be gradable. A full breakdown of these results is available in the Supplementary Materials.

When stratifying these results by grader profession, as shown in Table 2, there was no difference in overall accuracy between optometrists and ophthalmologists for the identification of referable retinopathy (85.7% and 87.5%, respectively, U = 24,440.0, *p* = 0.59) or when considering a grade score of 0 or 1 as correct (80.7% and 81.3%, respectively, U = 24,140.0, *p* = 0.88). Overall accuracy was no different when comparing grade scores of 0, (65.3% and 63.8%, respectively, U = 23,620.0, *p* = 0.74), although ophthalmologists were better at identifying proliferative diabetic retinopathy (66.7% and 86.7%, respectively, U = 720.0, *p* = 0.04).

**Figure 3:**
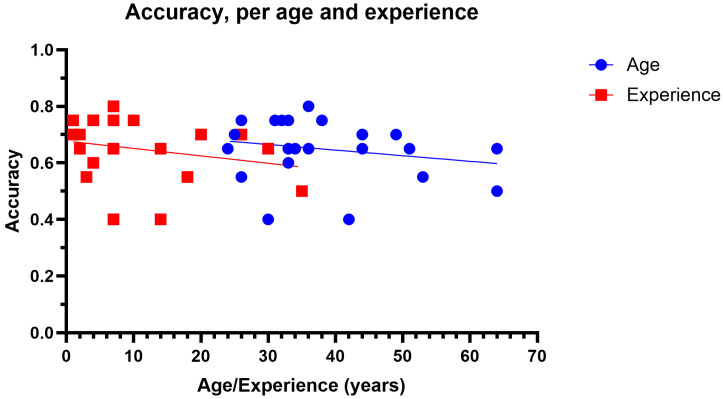
Accuracy as function of age (blue circles) and years of experience (red squares).

All additional analyses were conducted with a grade score of zero deemed correct and all other responses incorrect. No correlations were found between accuracy and years of experience (r^2^ = 0.06, *p* = 0.25) or accuracy and years of age (r^2^ = 0.04, *p* = 0.33), as shown in Figure 3.

Of the 40 images graded by participants, unanimous agreement on severity grade was obtained for only one image (mild NPDR). The inter-grader agreement was good overall (Krippendorff’s α = 0.818) [41], with higher agreement for image group 2 compared to group 1 (α = 0.836 and α = 0.755, respectively), which was not statistically significant. Similarly, there was moderate inter-grader agreement overall (α = 0.701) for the distinction between referable and non-referable diabetic retinopathy. Again, agreement was higher for group 2 compared to group 1 (α = 0.715 and α = 0.664, respectively), though this was not statistically significant. Agreement matrices illustrating inter-grader agreement are available in the Supplementary Materials (Figures S4 and S5).

### 3.2. Areas of Interest

**Figure 4:**
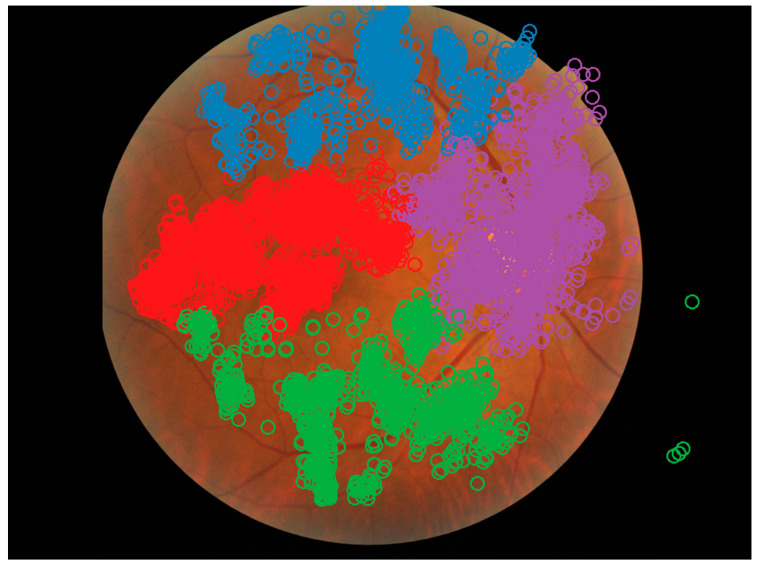
Example Hidden Markov Model from a single recording showing the optic disc, macula, superior, and inferior arcades as areas of interest. Each recorded gaze position is denoted as a circle, with the colour representing the cluster. Image from the DDR dataset [32].

Hidden Markov Model analysis found recorded gaze positions to be clustered around the optic disc, macula, superior temporal arcade, and inferior temporal arcade, with 173/460 (38%) of cases showing clusters around all four of these landmarks (66% of correct responses, 64% of incorrect responses). These five landmarks were used as AOIs for additional processing. An example analysis can be seen in Figure 4. As it was not possible to extract an exact macular shape and size from these data, the macula was defined as a circle of 5.5 mm in diameter centred on the foveola, with the foveola 4.76 mm from the centre of the optic disc according to work by Jonas et al. [42].

**Figure 5:**
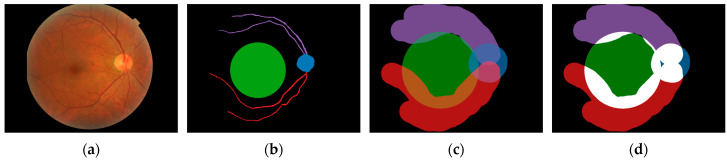
AOI annotations. (**a**) Original image from DDR dataset [32]. (**b**) Pixel-level annotations for optic disc (blue), superior temporal arcade (purple), inferior temporal arcade (red), and macula (green). (**c**) Expanded annotations based on eye tracker accuracy. (**d**) Overlapping AOIs are highlighted in white.

Pixel-level annotations were added for each image for each of the five AOIs. For the retinal arcades, arterioles and venules were annotated with the thicker vessel branch considered the main branch at each bifurcation, as described by Nowroozzadeh et al. [43]. An example of this annotation is shown in Figure 5, with areas of overlapping annotations highlighted.

### 3.3. Zoom Behaviour

No differences were found in the fraction of time spent at each zoom level overall. These data show that participants spent approximately one third of their time zoomed out (1.0×), one quarter of the time at a low level of zoom (1.5×), and one third of their time at a higher zoom level (3.38×). Participants rarely used moderate zoom (2.25×) or a zoom greater than 5.0×. A full breakdown of these data is available in the Supplementary Materials. When considering zoom levels per grade, correct responses for proliferative diabetic retinopathy were associated with the observer spending a higher fraction of time at 1.00× zoom compared to incorrect responses (0.39 and 0.20, respectively, U = 896.5, *p* = 0.02), and less time at 2.25× zoom (0.05 and 0.13, U = 452.5, *p* = 0.02). There were no other differences between correct and incorrect responses across grade and zoom levels.

When considering zoom behaviour per professional group, both optometrists and ophthalmologists spent over 90% of the time at zoom levels of 1.00×, 1.50×, and 3.38× zoom. Optometrists spent a lower fraction of time at 1.00× zoom for both correct (U = 7829.0, *p* < 0.01) and incorrect (U = 2799.5, *p* = 0.45) responses when compared to ophthalmologists. The reverse was found for 1.50× zoom, with optometrists spending less time at this level compared to ophthalmologists (U = 12,354.0, *p* < 0.01 and U = 3658.5, *p* = 0.02, respectively). At 3.38× zoom, optometrists spent more time at this level than ophthalmologists for correct responses (U = 11,436.0, *p* = 0.04), with no difference found for incorrect responses (U = 3168.5, *p* = 0.60). A full breakdown of these data is available in the Supplementary Materials.

### 3.4. Eye Movements

Overall, incorrect responses had longer dwell times compared to correct responses but showed no differences in other metrics. When considering individual AOIs, incorrect responses were associated with observers having more fixations, longer dwell times, and an increased total time in the macula compared to correct responses. The track length was also longer for incorrect responses (8098.77 px and 7633.96 px, U = 4,579,398.0, *p* < 0.01). There were no differences in total time spent at each zoom level between correct and incorrect groups. The full AOI fixation count, total visits, total time, and dwell time statistics are provided in the Supplementary Materials.

Additional differences were seen when considering these metrics per severity grade. For images with no retinopathy, correct responses were associated with less dwell time overall compared to incorrect responses (0.94 s and 0.98 s, U = 1,447,416.0, *p* = 0.01) and more dwell time in the superior arcade (0.90 s and 0.68 s, U = 98,329.5, *p* < 0.01).

Correct mild NPDR grading was associated with shorter dwell times overall (0.99 s and 1.12 s, U = 3,277,800.0, *p* < 0.01) and in the optic disc region (1.18 s and 1.47 s, U = 24,903.0, *p* < 0.01) and macula (1.00 s and 1.24 s, U = 545,269.5, *p* < 0.01). Correct responses were also associated with a shorter track length (7028.48 px and 7190.81 px, U = 411,678.0, *p* < 0.01).

Correct moderate NPDR responses were associated with longer dwell times overall (1.13 s and 1.04 s, U = 1,023,588.0, *p* < 0.01) and at the macula (1.10 s and 0.88 s, U = 171,859.5, *p* < 0.01). This differs from other severity grades, where these dwell times were shorter for correct responses.

Correct severe NPDR responses were associated with more AOI visits overall (7.85 and 6.60, U = 16,399.5, *p* = 0.02) but no differences when comparing individual AOIs. Dwell times were less overall (1.09 s and 1.39 s, U = 1,327,347.0, *p* < 0.01) and at the macula (1.04 s and 1.35 s, U = 231,894.0, *p* < 0.01) and optic disc (1.81 s and 2.64 s, U = 13,950.0, *p* = 0.01). Correct responses were also associated with lower track lengths (9742.18 px and 8327.37 px, U = 157,959.0, *p* < 0.01).

Correct proliferative DR grading was associated with less fixation counts overall (17.16 and 20.68, U = 18,007.0, *p* = 0.03) and in the inferior arcade (11.64 and 15.42, U = 544, *p* = 0.03), superior arcade (16.70 and 25.23, U = 627.0, *p* = 0.045) and macula (31.64 and 42.73, U = 532.0, *p* < 0.01). AOI visits were less overall (5.26 and 6.42, U = 15,715.0, *p* < 0.01) and at the macula (5.98 and 8.69, U = 451.5, *p* < 0.01). The time spent at each AOI was less overall (10.29 s and 15.17 s, U = 558.0, *p* = 0.01) although no differences were seen for individual AOIs. Dwell times were less in the superior arcade (0.89 s and 1.03 s, U = 133,150.5, *p* = 0.01) but more in the optic disc (1.75 s and 0.81 s, U = 34,002.0, *p* < 0.01). Track lengths were also less for this grade (7068.67 px and 10,117.62 px, U = 99,495.0, *p* < 0.01).

When comparing professional groups, optometrists had higher fixation counts, total visits per AOI and total time spent at each AOI compared to ophthalmologists, as shown in Table 3. This trend was seen for most AOIs for correct responses but was only significant for the macula and when considering all AOIs when responses were incorrect. Conversely, optometrists had lower dwell times overall compared to ophthalmologists, especially for incorrect responses. Track lengths were also longer for optometrists for correct (8482.30 px and 5947.85 px, U = 2,759,305.5, *p* < 0.01) and incorrect responses (8928.69 px and 6521.62 px, U = 876,591.0, *p* < 0.01).

### 3.5. Visual Search Patterns

**Figure 6:**
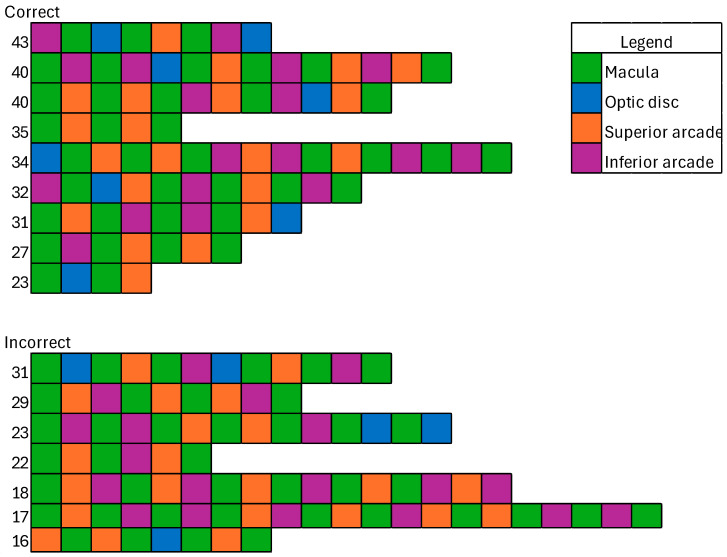
Fixation strings for correct and incorrect responses. Numbers represent the cluster size corresponding to this exemplar.

Visual search pattern analysis was undertaken considering those with a grade score of 0 (correct). The resulting fixation strings are shown in Figure 6. As the strings become excessively large for small clusters, representing outliers, the largest clusters have been included below, covering 75% of the responses, with the remaining data available in the Supplementary Materials. There was no difference in string length between correct and incorrect clusters (U = 169.0, *p* = 0.79).

## 4. Discussion

In this analysis of visual search behaviour during a photographic grading task for diabetic retinopathy, differences were found between correct and incorrect responses, including in visual search behaviours. When analysing the eye movement behaviour of participants, both overall and per severity grade, those whose grade score was zero had fewer fixations, fewer visits to each AOI, less time spent looking at each AOI, and shorter overall track lengths than those with incorrect responses. This suggests that pattern recognition alongside a structured search pattern may be a factor in grading efficiency. The increase in fixation count and total time spent in the macula region by correct respondents is in keeping with previous work by Murphy et al. [24] showing a high prevalence of diabetic retinopathy in this area, especially in the area temporal to the fovea.

**Figure 7:**
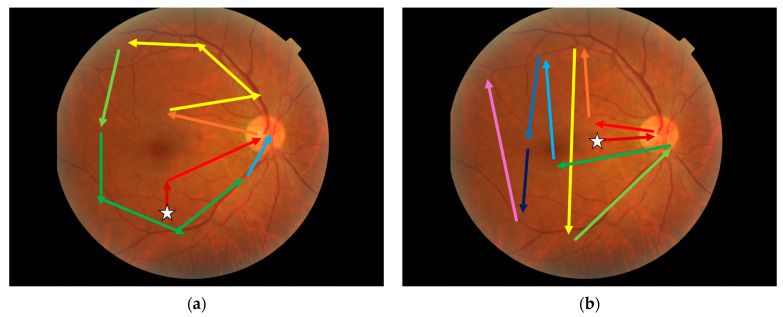
Visual search strategies extrapolated from Affinity Propagation clustering. (**a**) The most common correct string. (**b**) The most common incorrect string. White stars indicate the starting position. Retinal photograph from the DDR dataset [32].

The analysis of gaze data using Affinity Propagation shows differences in gaze behaviour between correct and incorrect respondents. While the fixation strings only show the order in which AOIs are visited, this can be extrapolated to suggest visual search strategies that follow this order. A possible interpretation of the most common correct and incorrect strings is shown in Figure 7. The pattern associated with correct grading shows little redundancy and perhaps indicates a conscious strategy of starting at the macula before looking around the superior arcade, then around to the inferior arcade, finishing at the optic disc. The pattern associated with incorrect response starts in the nasal region and moves temporally. This may suggest that a structured search pattern based on the anatomical features of the retina is superior to a saliency-based search when grading diabetic retinopathy from fundus photographs.

The results from this study may be useful for training healthcare professionals. It has been shown that educating optometrists to use the FORGE guidelines [16] can improve diagnostic performance in the context of glaucoma management [17], and a systematic review of using eye tracking for medical education noted that teaching expert gaze patterns can improve diagnostic speed. Therefore, developing a similar intervention for diabetic retinopathy is reasonable; however, such educational interventions require a theoretical grounding building upon current knowledge to be successful [30]. Teaching healthcare professionals or trainees to use a structured search strategy could leverage the results of this study alongside the pattern distribution [44,45] and pathophysiology of diabetic retinopathy features to improve grading performance. It is unclear whether this would be better targeted at practicing clinicians, who may have a stronger understanding of the disease, or trainees, to cement this knowledge as it is learned. Future work in this area is warranted.

To the authors’ knowledge, this is the first study to investigate zoom behaviour when screening fundus photographs for diabetic retinopathy. We found that graders spend approximately one third of their time zoomed out, one quarter of the time at a low level of zoom (1.5×), and one third of their time at a higher zoom level (3×). The zoom behaviour of graders did not differ between correct and incorrect groups but did appear to differ between the professions. Whether this reflects differences in age or experience between the groups in our cohort, differences in how these professions manage findings of pathology (referral vs. treatment) or other factors is unknown. Zoom steps were also arbitrarily chosen to be 150% of the previous zoom level, and it is unclear whether this design is optimal. More research in this area is warranted, with a larger cohort to exclude age or experience as potential confounders.

Hidden Markov Modelling suggests that graders tend to cluster their gazes around four anatomical features: the optic disc, macula, superior arcade, and inferior arcade, as shown in Figure 4. While it is expected that gaze data will cluster around these major anatomical landmarks, these data suggest that graders analyse each of these features separately. Whether this separation is seen in other examination contexts has not been investigated but may suggest natural gaze behaviour for clinicians assessing fundus images.

Our data show disagreement between graders in grading diabetic retinopathy using the International Clinical Diabetic Retinopathy Severity Scale [4], with an overall accuracy of 64.8%. Accuracy was similar between professions. This is in line with previous studies showing variability amongst graders [10,11,12]. Contrary to work by Sellahewa et al. [12], the disagreement was highest for moderate NPDR, with 37.5% of graders agreeing at this severity level (Supplementary Material Figure S3). These data do, however, agree with Sallam et al. [15], who found over- and under-grading in all severity grades. There was no difference in grading accuracy between optometrists and ophthalmologists overall; however, optometrists showed lower accuracy in detecting proliferative disease compared with ophthalmologists. The effect of this disagreement on clinical management is likely to be minimal as approximately 90% of referrable cases were correctly identified as such. This suggests that those who require assessment by an ophthalmologist will be seen, although perhaps not in the recommended timeframe. These findings indicate that there is room for improvement in inter-grader agreement in grading diabetic retinopathy.

Previous studies by Rangrej et al. [29] suggested longer dwell times and shorter track lengths for correct responses. Our study also showed shorter track lengths, which suggests a defined visual search strategy instead of saliency-driven search behaviour. However, our data identified shorter dwell times for correct responses. Our study also did not find any correlation between experience and grading accuracy. While the reasons for this are unclear, this may be due to the nature of the task; our study used images of all degrees of severities and required grading based on a grading scale, as required in clinical practice. The study by Rangrej et al. [29] only required images to be categorised as normal or abnormal, which does not require as much scrutiny of specific retinal abnormalities.

This study is not without limitations. Notably, this study used 45-degree fundus photographs without filters or additional imaging such as optical coherence tomography. Diabetic retinopathy frequently occurs outside of the region included in a 45-degree fundus photo [23,46]. It is not clear whether this gaze behaviour would be seen for images with a wider field of view or when assessing the retina clinically. Further research is recommended, to answer these questions.

Our participant population included more optometrists than ophthalmologists, due to the small pool of ophthalmologists with sub-specialty training in the area. While Table 3 does show some differences in gaze behaviour between these professions, this study found that using a structured approach to image assessment, rather than specific eye movements, was correlated with accurate grading. Hence, while additional ophthalmologist participants may reveal different eye movement patterns, the authors do not expect this key finding of the superiority of structured search to change.

The process used to create AOIs using eye tracking error parameters was only calculated once prior to data collection. This error is therefore overestimated when zooming in. Performing these calculations for each gaze record may provide more accurate data, though it may result in more noise when participants change zoom level. Future work comparing these two methods is warranted.

Our study also used data sourced from Chinese hospitals [32]. Replication using a more ethnically diverse dataset is warranted to confirm the findings of this study. Similarly, all participants work in Australia. The authors invite the replication of this study in other countries to investigate whether visual search behaviour differs based on practice location.

Despite these limitations, results from this study show differences in gaze behaviour in instances of correct grading compared to incorrect assessments that are not associated with clinical experience. This suggests that these data may help in developing a structured visual search strategy for diabetic retinopathy assessment to improve clinician diagnostic performance.

## Figures and Tables

**Table 1:** Participant information per group. Years of clinical experience—years since training completed.

	Age (Years)				Years of Clinical Experience			
	Mean	Min	Max	SD	Mean	Min	Max	SD
Ophthalmologist	48	33	64	11.40	16.5	1	35	11.86
Optometrist	33.9	24	51	8.13	8.5	1	30	8.55

**Table 2:** Grading accuracy (decimal) for participant groups. Values shown with standard deviations indicated in brackets. Bold rows indicate statistical significance.

	Severity	Optometrist	Ophthalmologist	Mann–Whitney U
Referable	All images	0.857 (±0.332)	0.875 (±0.332)	U = 23,560.0, *p* = 0.59
	Not referable	0.820 (±0.385)	0.861 (±0.348)	U = 5683.5, *p* = 0.43
	Referable	0.893 (±0.310)	0.923 (±0.268)	U = 5676.0, *p* = 0.47
Grade score = 0 or 1	All images	0.807 (±0.396)	0.813 (±0.392)	U = 23,860.0, *p* = 0.88
	No DR	0.983 (±0.129)	1.000 (±0.000)	U = 914.5, *p* = 0.49
	Mild NPDR	0.922 (±0.269)	0.896 (±0.309)	U = 2217.0, *p* = 0.60
	Moderate NPDR	0.578 (±0.499)	0.625 (±0.495)	U = 514.5, *p* = 0.71
	Severe NPDR	0.756 (±0.435)	0.625 (±0.495)	U = 610.5, *p* = 0.26
	**Proliferative DR**	**0.667 (±0.475)**	**0.867 (±0.346)**	**U = 720.0, *p* = 0.04**
Grade score = 0	All images	0.653 (±0.477)	0.638 (±0.482)	U = 24,380.0, *p* = 0.74
	No DR	0.800 (±0.403)	0.806 (±0.402)	U = 924, *p* = 0.95
	Mild NPDR	0.667 (±0.474)	0.688 (±0.468)	U = 2115.0, *p* = 0.47
	Moderate NPDR	0.444 (±0.503)	0.292 (±0.464)	U = 622.5, *p* = 0.22
	Severe NPDR	0.622 (±0.490)	0.458 (±0.509)	U = 628.5, *p* = 0.20
	**Proliferative DR**	**0.667 (±0.475)**	**0.867 (±0.346)**	**U = 720.0, *p* = 0.04**

**Table 3:** Fixation count, total visits, and total time statistics for each area of interest per professional group. Correct is defined as a grade score of zero. Values are mean ± standard deviation.

	**Fixation Count: Correct**			**Fixation Count: Incorrect**		
**AOI**	**Optometrist**	**Ophthalmologist**	**Mann–Whitney U**	**Optometrist**	**Ophthalmologist**	**Mann–Whitney U**
All	18.93 (±18.32)	14.60 (±16.09)	U = 275,265.5, *p* < 0.01	20.35 (±19.46)	16.80 (±17.56)	U = 79,749.5, *p* < 0.01
Arcade superior	17.04 (±12.43)	13.97 (±12.87)	U = 11,512.5, *p* < 0.01	19.52 (±17.62)	16.69 (±17.62)	U = 3435.5, *p* = 0.02
Arcade inferior	16.44 (±12.85)	11.34 (±9.80)	U = 11,743,5, *p* < 0.01	17.88 (±14.62)	14.47 (±12.55)	U = 3348.5, *p* = 0.08
Optic disc	10.46 (±8.23)	9.12 (±7.39)	U = 10,002.5, *p* = 0.08	10.14 (±9.44)	11.89 (±14.62)	U = 3046.5, *p* = 0.38
Macula	36.94 (±24.99)	28.54 (±23.29)	U = 12,411.0, *p* < 0.01	42.97 (±22.07)	30.84 (±22.93)	U = 3922.0, *p* < 0.01
	**Visits: Correct**			**Visits: Incorrect**		
**AOI**	**Optometrist**	**Ophthalmologist**	**Mann–Whitney U**	**Optometrist**	**Ophthalmologist**	**Mann–Whitney U**
All	6.36 (±4.21)	4.40 (±2.88)	U = 297,322.5, *p* < 0.01	6.30 (±4.03)	5.07 (±3.33)	U = 83,294.0, *p* < 0.01
Arcade superior	6.35 (±3.81)	4.56 (±3.09)	U = 12,252.0, *p* < 0.01	6.56 (±3.93)	5.35 (±3.30)	U = 3337.5, *p* = 0.05
Arcade inferior	6.16 (±4.10)	4.51 (±3.13)	U = 11,695.0, *p* < 0.01	6.04 (±3.94)	5.00 (±3.43)	U = 3342.5, *p* = 0.08
Optic disc	5.06 (±3.15)	3.70 (±2.15)	U = 11,065.5, *p* < 0.01	4.72 (±3.21)	3.94 (±2.86)	U = 3320.5, *p* = 0.06
Macula	8.52 (±4.72)	5.47 (±3.01)	U = 13,931.0, *p* < 0.01	8.80 (±4.41)	6.47 (±3.56)	U = 38,469.0, *p* < 0.01
	**Total Time (Seconds): Correct**			**Total Time (Seconds): Incorrect**		
**AOI**	**Optometrist**	**Ophthalmologist**	**Mann–Whitney U**	**Optometrist**	**Ophthalmologist**	**Mann–Whitney U**
All	6.00 (±5.88)	4.99 (±5.60)	U = 260,404.0, *p* < 0.01	6.71 (±7.05)	5.71 (±5.52)	U = 74,272.0, *p* = 0.14
Arcade superior	5.08 (±3.78)	4.24 (±3.65)	U = 11,092.0, *p* = 0.02	6.03 (±5.24)	4.94 (±4.33)	U = 3221.0, *p* = 0.13
Arcade inferior	5.13 (±3.83)	4.06 (±3.62)	U = 10,991.0, *p* = 0.01	6.00 (±5.15)	5.22 (±4.45)	U = 3122.0, *p* = 0.34
Optic disc	3.20 (±2.51)	2.99 (±2.31)	U = 9296.0, *p* = 0.53	3.17 (±3.18)	3.88 (±4.78)	U = 2833.0, *p* = 0.93
Macula	12.30 (±7.93)	10.15 (±8.33)	U = 11,788.0, *p* < 0.01	14.84 (±9.14)	10.87 (±6.91)	U = 3667.0, *p* < 0.01
	**Dwell Time (Seconds): Correct**			**Dwell Time (Seconds): Incorrect**		
**AOI**	**Optometrist**	**Ophthalmologist**	**Mann–Whitney U**	**Optometrist**	**Ophthalmologist**	**Mann–Whitney U**
All	1.00 (±0.98)	1.04 (±1.01)	U = 17,374,140.0, *p* = 0.02	1.06 (±1.13)	1.19 (±1.19)	U = 4,852,876.5, *p* < 0.01
Arcade superior	0.87 (±0.89)	0.90 (±0.95)	U = 1,110,123.0, *p* = 0.31	0.97 (±0.96)	0.81 (±0.73)	U = 364,432.5, *p* = 0.24
Arcade inferior	1.03 (±0.87)	1.09 (±0.88)	U = 856,611.0, *p* = 0.01	1.04 (±1.00)	1.23 (±1.02)	U = 211,855.5, *p* < 0.01
Optic disc	1.54 (±1.33)	1.56 (±1.46)	U = 205,146.0, *p* = 0.55	1.63 (±1.53)	1.99 (±1.81)	U = 49,374.0, *p* = 0.01
Macula	0.94 (±0.94)	0.95 (±0.90)	U = 3,043,863.0, *p* = 0.59	1.03 (±1.16)	1.16 (±1.16)	U = 852,885.0, *p* < 0.01

## Data Availability

Images used in this study were sourced from the publicly available DDR dataset [32]. All software for image processing and statistical analysis are available at https://github.com/tim-murphy/expert_dr_study (accessed on 10 June 2024).

## Supplementary Materials

The following supporting information can be downloaded at: https://www.mdpi.com/article/10.3390/jcm14093046/s1, Table S1: Practice image set; Table S2: Image set 1; Table S3: Image set 2; Table S4: Zoom statistics for correct and incorrect responses; Table S5: Zoom statistics split by professional group; Table S6: Fixation count, total visits, and total time statistics for each area of interest; Figure S1: Fixation strings for correct responses; Figure S2: Fixation strings for incorrect responses; Figure S3: Grade score distribution histograms; Figure S4: Inter-grader agreement matrix per severity grade; Figure S5: Inter-grader agreement matrix for referable status.

## References

[1] International Diabetes Federation (2021). IDF Diabetes Atlas.

[2] Flaxman S.R., Bourne R.R., Resnikoff S., Ackland P., Braithwaite T., Cicinelli M.V., Das A., Jonas J.B., Keeffe J., Kempen J.H. (2017). Global causes of blindness and distance vision impairment 1990–2020: A systematic review and meta-analysis. Lancet Glob. Health.

[3] McKay R., McCarty C.A., Taylor H.R. (2000). Diabetic retinopathy in Victoria, Australia: The Visual Impairment Project. Br. J. Ophthalmol..

[4] Wilkinson C., Ferris F.L., Klein R.E., Lee P.P., Agardh C.D., Davis M., Dills D., Kampik A., Pararajasegaram R., Verdaguer J.T. (2003). Proposed international clinical diabetic retinopathy and diabetic macular edema disease severity scales. Ophthalmology.

[5] Early Treatment Diabetic Retinopathy Study Research Group (1991). Grading Diabetic Retinopathy from Stereoscopic Color Fundus Photographs—An Extension of the Modified Airlie House Classification. Ophthalmology.

[6] Mitchell P., Foran S., Wong T.Y., Chua B., Patel I., Ojaimi E., Foran J. (2008). Guidelines for the Management of Diabetic Retinopathy.

[7] Wong T.Y., Sun J., Kawasaki R., Ruamviboonsuk P., Gupta N., Lansingh V.C., Maia M., Mathenge W., Moreker S., Muqit M.M. (2018). Guidelines on Diabetic Eye Care. Ophthalmology.

[8] Public Health England (2021). Diabetic Eye Screening Standards Valid for Data Collected from 1 April 2019 [Internet]. https://www.gov.uk/government/publications/diabetic-eye-screening-programme-standards/diabetic-eye-screening-standards-valid-for-data-collected-from-1-april-2019.

[9] Optometry Australia (2018). Clinical Guideline: Examination and Management of Patients with Diabetes [Internet]. https://www.optometry.org.au/wp-content/uploads/Professional_support/Guidelines/clinical_guideline_diabetes_revised_sept_2018_final_designed.pdf.

[10] Schmid K.L., Swann P.G., Pedersen C., Schmid L.M. (2002). The detection of diabetic retinopathy by Australian optometrists. Clin. Exp. Optom..

[11] Krause J., Gulshan V., Rahimy E., Karth P., Widner K., Corrado G.S., Peng L., Webster D.R. (2018). Grader Variability and the Importance of Reference Standards for Evaluating Machine Learning Models for Diabetic Retinopathy. Ophthalmology.

[12] Idris I., Sellahewa L., Simpson C., Maharajan P., Duffy J. (2014). Grader agreement, and sensitivity and specificity of digital photography in a community optometry-based diabetic eye screening program. Clin. Ophthalmol..

[13] Teoh C.S., Wong K.H., Xiao D., Wong H.C., Zhao P., Chan H.W., Yuen Y.S., Naing T., Yogesan K., Koh V.T.C. (2023). Variability in Grading Diabetic Retinopathy Using Retinal Photography and Its Comparison with an Automated Deep Learning Diabetic Retinopathy Screening Software. Healthcare.

[14] Fahadullah M., Memon N.A., Salim S., Ahsan S., Fahim M.F., Mumtaz S.N., Shaikh S.A., Memon M.S. (2019). Diagnostic accuracy of non-mydriatic fundus camera for screening of diabetic retinopathy: A hospital based observational study in Pakistan. J. Pak. Med. Assoc..

[15] Sallam A., Scanlon P.H., Stratton I.M., Jones V., Martin C.N., Brelen M., Johnston R.L. (2011). Agreement and reasons for disagreement between photographic and hospital biomicroscopy grading of diabetic retinopathy. Diabet. Med..

[16] Fingeret M., Medeiros F.A., Susanna R., Weinreb R.N. (2005). Five rules to evaluate the optic disc and retinal nerve fiber layer for glaucoma. Optom.-J. Am. Optom. Assoc..

[17] Yoshioka N., Wong E., Kalloniatis M., Yapp M., Hennessy M.P., Agar A., Healey P.R., Hayen A., Zangerl B. (2015). Influence of education and diagnostic modes on glaucoma assessment by optometrists. Ophthalmic Physiol. Opt..

[18] Leong J., Nicolaou M., Emery R., Darzi A., Yang G.-Z. (2007). Visual search behaviour in skeletal radiographs: A cross-speciality study. Clin. Radiol..

[19] Uchida S., Hiraoka S.-I., Kawamura K., Sakamoto K., Akiyama R., Tanaka S. (2023). Machine Learning Analysis of Gaze Data for Enhanced Precision in Diagnosing Oral Mucosal Diseases. J. Clin. Med..

[20] Krupinski E.A., Chao J., Hofmann-Wellenhof R., Morrison L., Curiel-Lewandrowski C. (2014). Understanding Visual Search Patterns of Dermatologists Assessing Pigmented Skin Lesions Before and After Online Training. J. Digit. Imaging.

[21] Drew T., Lavelle M., Kerr K.F., Shucard H., Brunyé T.T., Weaver D.L., Elmore J.G. (2021). More scanning, but not zooming, is associated with diagnostic accuracy in evaluating digital breast pathology slides. J. Vis..

[22] Verma A., Alagorie A.R., Ramasamy K., van Hemert J., Yadav N., Pappuru R.R., Tufail A., Nittala M.G., Sadda S.R., Indian Retina Research Associates (IRRA) (2020). Distribution of peripheral lesions identified by mydriatic ultra-wide field fundus imaging in diabetic retinopathy. Graefe’s Arch. Clin. Exp. Ophthalmol..

[23] Silva P.S., Cavallerano J.D., Sun J.K., Soliman A.Z., Aiello L.M., Aiello L.P. (2013). Peripheral Lesions Identified by Mydriatic Ultrawide Field Imaging: Distribution and Potential Impact on Diabetic Retinopathy Severity. Ophthalmology.

[24] Murphy T.I., Douglass A.G., van Wijngaarden P., Armitage J.A. (2024). Programmatically Localizing Diabetic Retinopathy Features in 45-Degree Retinal Photographs Using Anatomical Colocation. J. Clin. Med..

[25] Coutrot A., Hsiao J.H., Chan A.B. (2018). Scanpath modeling and classification with hidden Markov models. Behav. Res. Methods.

[26] Al-Moteri M.O., Symmons M., Plummer V., Cooper S. (2017). Eye tracking to investigate cue processing in medical decision-making: A scoping review. Comput. Hum. Behav..

[27] Brockmole J.R., Henderson J.M. (2006). Short Article: Recognition and Attention Guidance during Contextual Cueing in Real-World Scenes: Evidence from Eye Movements. Q. J. Exp. Psychol..

[28] O’Neill E.C., Kong Y.X.G., Connell P.P., Ni Ong D., Haymes S.A., Coote M.A., Crowston J.G. (2011). Gaze Behavior among Experts and Trainees during Optic Disc Examination: Does How We Look Affect What We See?. Investig. Opthalmol. Vis. Sci..

[29] Rangrej S.B., Sivaswamy J., Srivastava P. (2018). Scan, dwell, decide: Strategies for detecting abnormalities in diabetic retinopathy. PLoS ONE.

[30] Kok E.M., Jarodzka H. (2017). Before your very eyes: The value and limitations of eye tracking in medical education. Med. Educ..

[31] Rajalakshmi R., Prathiba V., Arulmalar S., Usha M. (2021). Review of retinal cameras for global coverage of diabetic retinopathy screening. Eye.

[32] Li T., Gao Y., Wang K., Guo S., Liu H., Kang H. (2019). Diagnostic assessment of deep learning algorithms for diabetic retinopathy screening. Inf. Sci..

[33] Murphy T.I., Abel L.A., Armitage J.A., Douglass A.G. (2024). Effects of tracker location on the accuracy and precision of the Gazepoint GP3 HD for spectacle wearers. Behav. Res. Methods.

[34] Bellemo V., Lim Z.W., Lim G., Nguyen Q.D., Xie Y., Yip M.Y.T., Hamzah H., Ho J., Lee X.Q., Hsu W. (2019). Artificial intelligence using deep learning to screen for referable and vision-threatening diabetic retinopathy in Africa: A clinical validation study. Lancet Digit. Health.

[35] Krippendorff K. (1970). Estimating the Reliability, Systematic Error and Random Error of Interval Data. Educ. Psychol. Meas..

[36] Heeman J., Theeuwes J., Van der Stigchel S. (2014). The time course of top-down control on saccade averaging. Vis. Res..

[37] Frey B.J., Dueck D. (2007). Clustering by Passing Messages Between Data Points. Science.

[38] Levenshtein V.I. (1966). Binary codes capable of correcting deletions, insertions, and reversals. Soviet Physics Doklady.

[39] Wedel M., Yan J., Smith P., Siegel E., Li H.A. (2014). A Hidden Markov Model to Identify Regions of Interest from Eye Movements, with an Application to Nodule Detection in Chest X-Rays. SSRN Electron. J..

[40] Wang Y.T., Tadarati M., Wolfson Y., Bressler S.B., Bressler N.M. (2016). Comparison of Prevalence of Diabetic Macular Edema Based on Monocular Fundus Photography *vs.* Optical Coherence Tomography. JAMA Ophthalmol..

[41] Marzi G., Balzano M., Marchiori D. (2024). K-Alpha Calculator–Krippendorff’s Alpha Calculator: A user-friendly tool for computing Krippendorff’s Alpha inter-rater reliability coefficient. MethodsX.

[42] Jonas R.A., Wang Y.X., Yang H., Li J.J., Xu L., Panda-Jonas S., Jonas J.B. (2015). Optic Disc—Fovea Distance, Axial Length and Parapapillary Zones. The Beijing Eye Study 2011. PLoS ONE.

[43] Nowroozzadeh M.H., Moshksar S., Azimi A., Rasti A., Sedaghat A. (2022). Comparison of retinal vascular arcade trajectory between eyes with an idiopathic macular hole and the healthy fellow eye. Int. Ophthalmol..

[44] Murphy T., Armitage J., van Wijngaarden P., Abel L., Douglass A. (2024). Unmasking visual search: An objective framework for grouping eye tracking data. Investig. Ophthalmol. Vis. Sci..

[45] Munuera-Gifre E., Saez M., Juvinyà-Canals D., Rodríguez-Poncelas A., Barrot-De-La–Puente J., Franch-Nadal J., Romero-Aroca P., Barceló M.A., Coll-De-Tuero G. (2020). Analysis of the location of retinal lesions in central retinographies of patients with Type 2 diabetes. Acta Ophthalmol..

[46] Li X., Xie J., Zhang L., Cui Y., Zhang G., Wang J., Zhang A., Chen X., Huang T., Meng Q. (2020). Differential distribution of manifest lesions in diabetic retinopathy by fundus fluorescein angiography and fundus photography. BMC Ophthalmol..

