# Supplementary material for: Examining the Visual Search Behaviour of Experts When Screening for the Presence of Diabetic Retinopathy in Fundus Images
Source: J Clin Med. 2025 Apr 28;14(9):3046. doi: 10.3390/jcm14093046 (PMC12073068; doi:10.3390/jcm14093046)
Supplement: Supplementary file 1 [file jcm-14-03046-s001.zip › jcm-3599218-supplementary.pdf]

Supplementary material

Image sets

The following image sets were used by the software. All images were sourced from the DDR dataset.[1] These data include disease severity, graded against the International Clinical Diabetic Retinopathy Severity Scale.[2]

Table S1. *Practice image set.*

Image name	Diabetic retinopathy severity grade
007-0034-000.jpg	No diabetic retinopathy
007-0059-000.jpg	No diabetic retinopathy
007-0089-000.jpg	No diabetic retinopathy
007-2339-100.jpg	Moderate NPDR
007-2390-100.jpg	Moderate NPDR
007-2391-100.jpg	Moderate NPDR
007-2453-100.jpg	Moderate NPDR
007-2457-100.jpg	Mild NPDR
007-2466-100.jpg	Moderate NPDR
007-3485-200.jpg	Moderate NPDR
007-3638-200.jpg	Moderate NPDR
007-3729-200.jpg	Moderate NPDR
007-3792-200.jpg	Moderate NPDR
007-4032-200.jpg	Moderate NPDR
007-4258-200.jpg	Moderate NPDR
007-4433-200.jpg	Proliferative DR
007-5435-300.jpg	Moderate NPDR
007-6160-300.jpg	Severe NPDR
007-6545-400.jpg	Proliferative DR
20170514222317943.jpg	Proliferative DR
20170519153500194.jpg	Mild NPDR

Table S2. *Image set 1.*

Image name	Diabetic retinopathy severity grade
007-0028-000.jpg	No diabetic retinopathy
007-0055-000.jpg	No diabetic retinopathy
007-0142-000.jpg	No diabetic retinopathy
007-0323-000.jpg	No diabetic retinopathy
007-1774-100.jpg	Mild NPDR
007-2252-100.jpg	Moderate NPDR
007-2469-100.jpg	Mild NPDR
007-2477-100.jpg	Moderate NPDR
007-2840-100.jpg	Mild NPDR
007-4250-200.jpg	Severe NPDR
007-4850-300.jpg	Severe NPDR
007-5457-300.jpg	Severe NPDR
007-6320-400.jpg	Proliferative DR
007-6573-400.jpg	Proliferative DR
007-7146-400.jpg	Proliferative DR
007-7235-400.jpg	Proliferative DR
20170502092649506.jpg	Moderate NPDR
20170518171333730.jpg	Mild NDPR
20170519153000176.jpg	Severe NPDR
20170521094743135.jpg	Moderate NPDR

Table S3. *Image set 2.*

Image name	Diabetic retinopathy severity grade
007-0051-000.jpg	No diabetic retinopathy
007-0079-000.jpg	No diabetic retinopathy
007-0321-000.jpg	No diabetic retinopathy
007-1811-100.jpg	Mild NPDR
007-2403-100.jpg	Moderate NPDR
007-2705-100.jpg	Mild NPDR
007-2763-100.jpg	Moderate NPDR
007-2841-100.jpg	Mild NPDR
007-4290-200.jpg	Severe NPDR
007-4991-300.jpg	Severe NPDR
007-6520-400.jpg	Proliferative DR
007-7017-400.jpg	Proliferative DR
007-7175-400.jpg	Proliferative DR
007-7265-400.jpg	Proliferative DR
20170228231807286.jpg	No diabetic retinopathy
20170511224838938.jpg	Moderate NPDR
20170512231007763.jpg	Severe NPDR
20170525111826224.jpg	Moderate NPDR
20170609154552424.jpg	Mild NPDR
20170622080837660.jpg	Severe NPDR

Fixation string clusters

Below are the full set of clusters generated using Affinity Propagation for correct and incorrect responses. The full list was not included in the main manuscript as the smaller clusters are excessively large and are not relevant for the processing undertaken in this study.

Correct

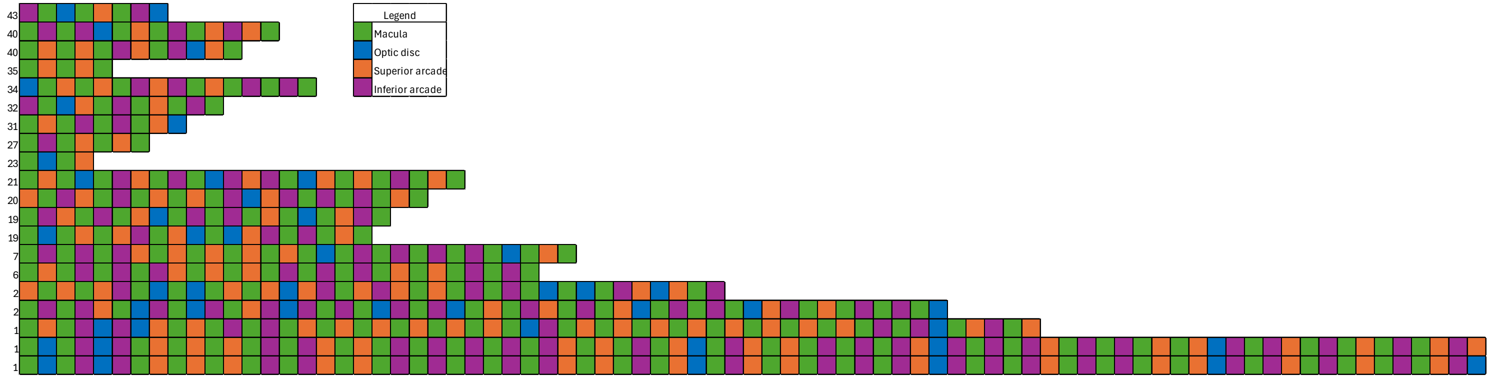

Figure S1. *Fixation strings for correct responses. Numbers represent the cluster size corresponding to this exemplar.*

Incorrect

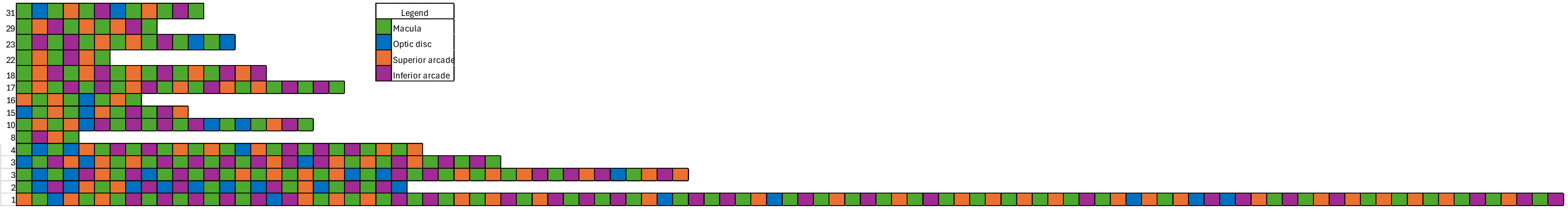

Figure S2. *Fixation strings for incorrect responses. Numbers represent the cluster size corresponding to this exemplar.*

Grade score distribution

Below is the distribution of grade scores per disease severity.

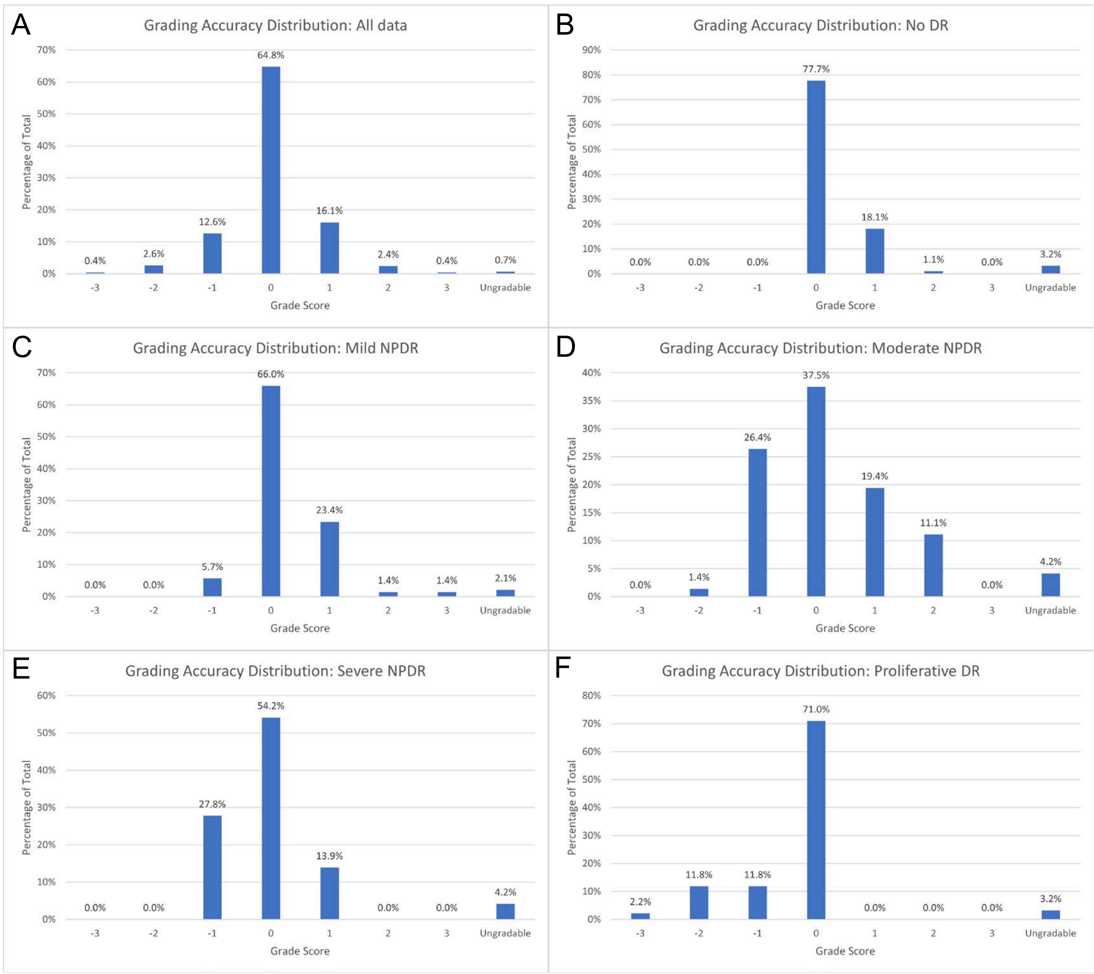

Figure S3. *Distribution of grade scores for A: all data, B: images with no diabetic retinopathy, C: images of mild NPDR, D: images of moderate NPDR, E: images of severe NPDR, and F: images of proliferative DR.*

Inter-grader agreement matrix

The following matrices illustrate inter-grader agreement for the two image groups, using both the International Clinical Diabetic Retinopathy Severity Scale[2] and a referable/not referable binary scale.

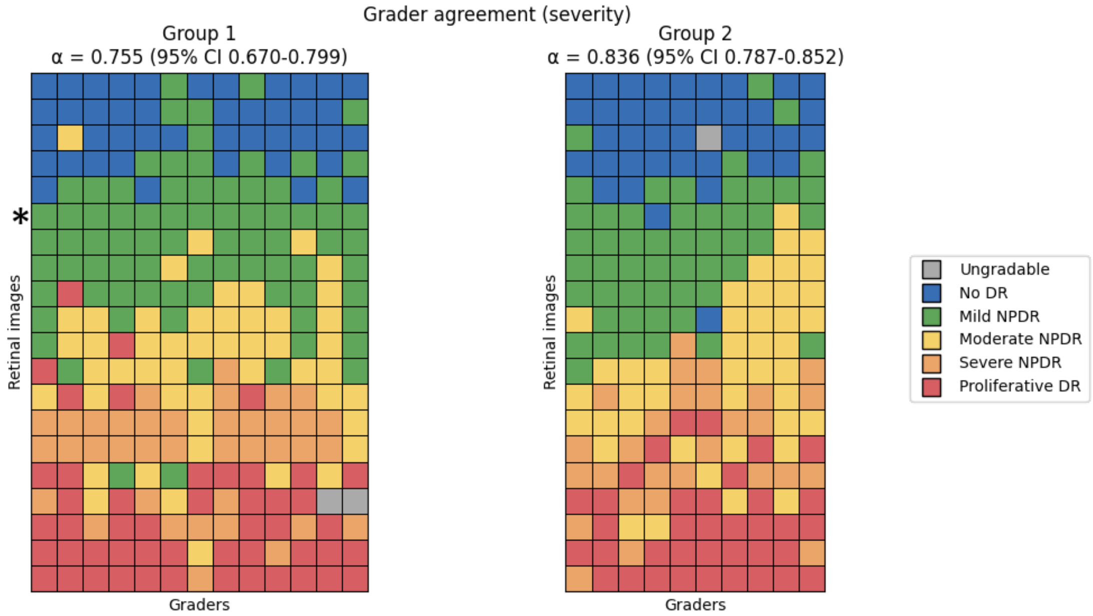

Figure S4. *Inter-grader agreement of diabetic retinopathy grading, with columns representing participants and rows representing images. Cell colors indicate the severity grade given to an image by a participant. Asterisks indicates unanimous agreement.*

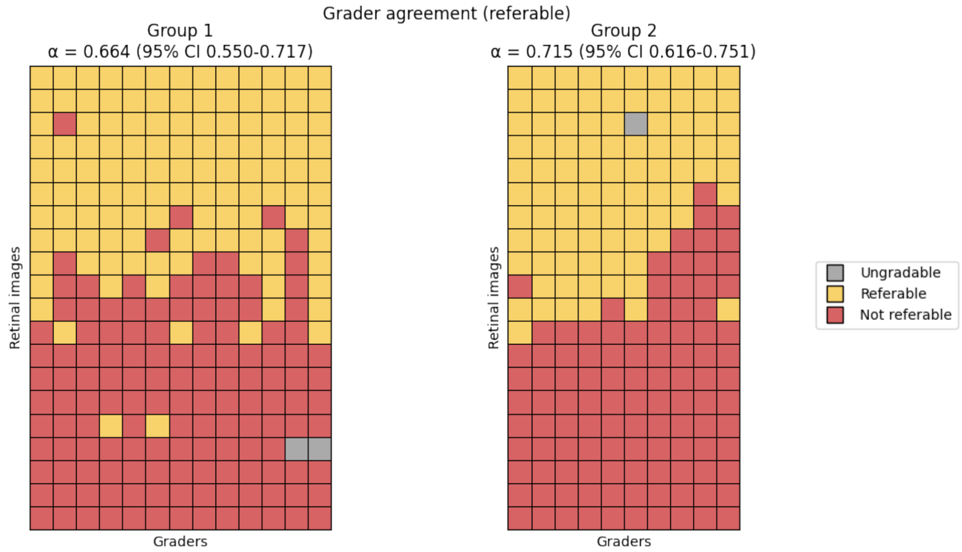

Figure S5. *Inter-grader agreement of referable diabetic retinopathy, with columns representing participants and rows representing images.*

Zoom statistics

The following tables contain statistics on zoom behaviors of participants.

Table S4. *Zoom statistics for correct and incorrect responses. Correct is defined as a grade score of zero.*

Zoom (fraction of total time)			
Zoom level	Correct mean (± SD)	Incorrect mean (± SD)	Mann-Whitney U
1.00 x	0.35 (± 0.32)	0.33 (±0.33)	U = 25412.0, p = 0.35
1.50 x	0.28 (± 0.29)	0.28 (± 0.28)	U = 23517.0, p = 0.65
2.25 x	0.05 (± 0.10)	0.07 (± 0.15)	U = 23527.0, p = 0.62
3.38 x	0.31 (± 0.29)	0.30 (± 0.28)	U = 24363.0, p = 0.87
> 5.00 x	0.01 (± 0.05)	0.01 (± 0.02)	U = 24743.0, p = 0.49

Table S5. *Zoom statistics for correct and incorrect responses per professional group. Correct is defined as a grade score of zero.*

**Zoom (fraction of total time): correct**			
**Zoom level**	**Optometrist mean (± SD)**	**Ophthalmologist mean (± SD)**	**Mann-Whitney U**
1.00 x	0.30 (± 0.28)	0.45 (± 0.37)	U = 7829.0, p < 0.01
1.50 x	0.30 (± 0.27)	0.23 (± 0.31)	U = 12354.0, p < 0.01
2.25 x	0.05 (± 0.10)	0.04 (± 0.10)	U = 12507.0, p < 0.01
3.38 x	0.32 (± 0.29)	0.28 (± 0.30)	U = 11436.0, p = 0.04
> 5.00 x	0.02 (± 0.05)	0.00 (± 0.01)	U = 12072.0, p < 0.01
**Zoom (fraction of total time): incorrect**			
**Zoom level**	**Zoom level**	**Zoom level**	**Zoom level**
1.00 x	0.30 (± 0.30)	0.39 (± 0.37)	U = 2799.5, p = 0.45
1.50 x	0.31 (± 0.26)	0.25 (± 0.32)	U = 3658.5, p = 0.02
2.25 x	0.08 (± 0.14)	0.04 (± 0.16)	U = 4081.5, p < 0.01
3.38 x	0.30 (± 0.26)	0.31 (± 0.32)	U = 3168.5, p = 0.60
> 5.00 x	0.01 (± 0.02)	0.00 (± 0.01)	U = 3541.0, p < 0.01

Fixation statistics

The following tables contain fixation statistics.

Table S6. *Fixation count, total visits and total time statistics for each area of interest. Correct is defined as a grade score of zero.*

**Fixation count**			
**AOI**	**Correct mean (± SD)**	**Incorrect mean (± SD)**	**Mann-Whitney U**
All	17.48 (± 17.71)	19.12 (± 18.89)	U = 538006.5, p = 0.06
Arcade superior	16.01 (± 12.64)	18.55 (± 17.62)	U = 21217.0, p = 0.16
Arcade inferior	14.71 (± 12.13)	16.70 (± 13.99)	U = 20844.5, p = 0.13
Optic disc	10.02 (± 7.98)	10.74 (± 11.46)	U = 23158.0, p = 0.57
Macula	34.10 (± 24.72)	38.77 (± 23.04)	U = 20107.0, p = 0.01
**Visits**			
**AOI**	**Correct mean (± SD)**	**Incorrect mean (± SD)**	**Mann-Whitney U**
All	5.70 (± 3.93)	5.88 (± 3.85)	U = 543272.0, p = 0.14
Arcade superior	5.75 (± 3.68)	6.15 (± 3.76)	U = 21485.5, p = 0.23
Arcade inferior	5.60 (± 3.87)	5.68 (± 3.79)	U = 22382.5, p = 0.74
Optic disc	4.61 (± 2.93)	4.46 (± 3.11)	U = 23502.0, p = 0.40
Macula	7.49 (± 4.45)	7.99 (± 4.27)	U = 21523.0, p = 0.13

		Total time (seconds)	
**AOI**	**Correct mean (± SD)**	**Incorrect mean (± SD)**	**Mann-Whitney U**
All	5.66 (± 5.81)	6.37 (± 6.58)	U = 538447.5, p = 0.07
Arcade superior	4.79 (± 3.75)	5.66 (± 4.96)	U = 21126.0, p = 0.14
Arcade inferior	4.77 (± 3.79)	5.73 (± 4.92)	U = 20708.5, p = 0.11
Optic disc	3.13 (± 2.45)	3.41 (± 3.80)	U = 23487.0, p = 0.41
Macula	11.57 (± 8.12)	13.47 (± 8.62)	U = 20205.0, p = 0.01
		**Dwell time (seconds)**	
**AOI**	**Correct mean (± SD)**	**Incorrect mean (± SD)**	**Mann-Whitney U**
All	1.01 (± 0.99)	1.10 (± 1.15)	U = 47335342.5, p = 0.04
Arcade superior	0.88 (± 0.91)	0.92 (± 0.90)	U = 3172630.5, p = 0.35
Arcade inferior	1.05 (± 0.88)	1.10 (± 1.01)	U = 2336314.5, p = 0.65
Optic disc	1.55 (± 1.37)	1.77 (± 1.65)	U = 459900.0, p = 0.12
Macula	0.94 (± 0.93)	1.07 (± 1.16)	U = 8279163.0, p = 0.01

References

1. Li T, Gao Y, Wang K, Guo S, Liu H, Kang H. Diagnostic assessment of deep learning algorithms for diabetic retinopathy screening. Inf Sci (Ny). 2019 Oct 1;501:511–22.

2. Wilkinson CP, Ferris FL, Klein RE, Lee PP, Agardh CD, Davis M, et al. Proposed international clinical diabetic retinopathy and diabetic macular edema disease severity scales. Ophthalmology. 2003 Jan 1;110(9):1677–82.
